# Methodology for Conducting Post-Marketing Surveillance of Software as a Medical Device Based on Artificial Intelligence Technologies

**DOI:** 10.17691/stm2022.14.5.02

**Published:** 2022-09-29

**Authors:** V.V. Zinchenko, K.M. Arzamasov, S.F. Chetverikov, A.V. Maltsev, V.P. Novik, E.S. Akhmad, D.E. Sharova, A.E. Andreychenko, A.V. Vladzymyrskyy, S.P. Morozov

**Affiliations:** Head of the Clinical and Technical Trials Sector, Department of Innovative Technologies; Research and Practical Clinical Center for Diagnostics and Telemedicine Technologies of the Moscow Health Care Department, Bldg 1, 24 Petrovka St., Moscow, 127051, Russia; Head of the Department of Medical Informatics, Radiomics and Radiogenomics; Research and Practical Clinical Center for Diagnostics and Telemedicine Technologies of the Moscow Health Care Department, Bldg 1, 24 Petrovka St., Moscow, 127051, Russia; Engineer, Sector of the Development of Systems for the Implementation of Medical Intelligent Technologies, Department of Medical Informatics, Radiomics and Radiogenomics; Research and Practical Clinical Center for Diagnostics and Telemedicine Technologies of the Moscow Health Care Department, Bldg 1, 24 Petrovka St., Moscow, 127051, Russia; Senior Researcher, Sector of Clinical and Technical Trials, Department of Innovative Technologies; Research and Practical Clinical Center for Diagnostics and Telemedicine Technologies of the Moscow Health Care Department, Bldg 1, 24 Petrovka St., Moscow, 127051, Russia; Researcher, Sector of the Development of Systems for the Implementation of Medical Intelligent Technologies, Department of Medical Informatics, Radiomics and Radiogenomics; Research and Practical Clinical Center for Diagnostics and Telemedicine Technologies of the Moscow Health Care Department, Bldg 1, 24 Petrovka St., Moscow, 127051, Russia; Researcher, Sector of Clinical and Technical Trials, Department of Innovative Technologies; Research and Practical Clinical Center for Diagnostics and Telemedicine Technologies of the Moscow Health Care Department, Bldg 1, 24 Petrovka St., Moscow, 127051, Russia; Deputy Head of the Department of Innovative Technologies; Research and Practical Clinical Center for Diagnostics and Telemedicine Technologies of the Moscow Health Care Department, Bldg 1, 24 Petrovka St., Moscow, 127051, Russia; Leading Researcher, Department of Medical Informatics, Radiomics and Radiogenomics; Research and Practical Clinical Center for Diagnostics and Telemedicine Technologies of the Moscow Health Care Department, Bldg 1, 24 Petrovka St., Moscow, 127051, Russia; Deputy Director for Science; Research and Practical Clinical Center for Diagnostics and Telemedicine Technologies of the Moscow Health Care Department, Bldg 1, 24 Petrovka St., Moscow, 127051, Russia; Professor, Radiologist; Research and Practical Clinical Center for Diagnostics and Telemedicine Technologies of the Moscow Health Care Department, Bldg 1, 24 Petrovka St., Moscow, 127051, Russia

**Keywords:** artificial intelligence, medical software, a post-marketing surveillance

## Abstract

**The aim of the study** was to develop a methodology for conducting post-registration clinical monitoring of software as a medical device based on artificial intelligence technologies (SaMD-AI).

**Materials and Methods:**

The methodology of post-registration clinical monitoring is based on the requirements of regulatory legal acts issued by the Board of the Eurasian Economic Commission. To comply with these requirements, the monitoring involves submission of the review of adverse events reports, the review of developers’ routine reports on the safety and efficiency of SaMD-AI, and the assessment of the system for collecting and analyzing developers’ post-registration data on the safety and efficiency of medical devices. The methodology was developed with regard to the recommendations of the International Medical Device Regulators Forum and the documents issued by the Food and Drug Administration (USA). Field-testing of this methodology was carried out using SaMD-AI designed for diagnostic imaging.

**Results:**

The post-registration monitoring of SaMD-AI consists of three key stages: collecting user feedback, technical monitoring and clinical validation. Technical monitoring involves routine evaluation of SaMD-AI output data quality to detect and remove flaws in a timely manner, and to secure the product stability. Major outcomes include an ordered list of technical flaws in SaMD-AI and their classification using evidence from diagnostic imaging studies. The application of this methodology resulted in a gradual reduction in the number of studies with flaws due to timely improvements in artificial intelligence algorithms: the number of flaws decreased to 5% in various aspects during subsequent testing. Clinical validation confirmed that SaMD-AI is capable of producing clinically meaningful outputs related to its intended use within the functionality determined by the developer. The testing procedure and the baseline testing framework were established during the field testing.

**Conclusion:**

The developed methodology will ensure the safety and efficiency of SaMD-AI taking into account its specifics as intangible medical devices. The methodology presented in this paper can be used by SaMD-AI developers to plan and carry out the post-registration clinical monitoring.

## Introduction

Monitoring of medical device (MD) safety, being a part of state control over MD circulation, plays an important role both worldwide [1-3] and in Russia [4, 5]. Such monitoring is aimed at ensuring the safety and efficiency of medical devices in real-world practice. The use of high-risk (class III) MD demands tighter monitoring requirements. Thus, the Decision of the Board of the Eurasian Economic Commission [6] determined that the monitoring of risk class III medical devices must be carried out annually for three years after obtaining a registration certificate, even if there are no adverse events or risks from the product. This type of monitoring is called a post-marketing surveillance (PSM).

SaMD-AI (software as a medical device based on artificial intelligence technologies) utilizes technical solutions that simulate human cognition and deliver results that are at least comparable to that of humans [7, 8]. SaMD-AI requires special monitoring during operation, since it lacks data interpretability. This may cause bias when used on a population different from that used in machine learning, etc. [9, 10]. Besides, unlike SaMD without AI technologies, it may include deep neural networks with continuous (self-)learning [11]. In this regard, SaMD-AI is assigned to a high-risk class — сlass III MD [12], which makes annual PSM mandatory for three years following the registration. This procedure should increase the user confidence in SaMD-AI, and ensure safety and efficiency throughout the total product life cycle [13-15].

However, the requirements and recommendations for PSM in published documents are but generic for MD and fail to embrace the unique features of SaMD-AI which requires a special approach to such monitoring. The latter must ensure both the effectiveness and safety of the finished product in routine clinical practice.

The approaches to setting requirements to the SaMD-AI developers to ensure the product efficiency and safety are well-known (see Good Machine Learning Practice [16]). In order to determine possible changes in SaMD-AI, the developers create documents titled “Configuration and change management plan” as defined by GOST R IEC 62304—2013, or “Predetermined Change Control Plan” as mandated by the Food Drug Administration documentation (FDA, USA) [16].

During PSM, it is important to attest the safety and efficiency of using SaMD-AI in routine clinical practice, and to collect and analyze user feedback. And although the PSM methodology for MD, which includes the validation and verification mechanisms and aggregation of user feedback, has already been developed and debugged, still SaMD-AI will greatly benefit from its own monitoring methodology.

**The aim of this study** was to develop a methodology for post-marketing surveillance of the SaMD-AI, and to perform further field-testing.

## Materials and Methods

The methodology of PSM of SaMD-AI performance within the framework of national regulatory area is based on the requirements approved by the Decision of the Board of the Eurasian Economic Commission [6]. The Decision states, that the monitoring must include the review of the adverse event reports, the review of routine reports from the developers on the safety and efficiency of SaMD-AI, and the assessment of the system for collecting and analyzing developers’ post-registration data on safety and efficiency of MD. PSM is carried out in accordance with the plan drawn up by the SaMD-AI developer during the development stage. This plan must include goals and objectives, as well as a PSM scheme with a detailed rationale of the methods used, demographics of the study subjects, inclusion/exclusion criteria, etc.

In addition, the recommendations of foreign professional communities and regulatory bodies were analyzed. The recommendations of the International Medical Device Regulators Forum (IMDRF) developed for software as an MD [14, 17], FDA documents [11, 18], as well as additional literature [2, 13, 15, 19, 20] were also considered.

Taking into account the recommendations from the above sources, several stages of the PSM have been identified.

1. Feedback. In order to analyze reports on adverse events, a feedback collection and analysis system must be organized.
2. Technical monitoring. In order to prepare routine reports on SaMD-AI safety, the developers must perform product inspections for laws that could lead to adverse events.
3. Clinical validation. During the real-world testing of the SaMD-AI effectiveness, it is necessary to confirm the accuracy metrics of the product by testing it on a data set.

The proposed PSM methodology was tested as part of the experiment on the use of innovative technologies in the field of computer vision for the analysis of medical images and further application in the healthcare system of Moscow (hereinafter referred to as the Experiment) [21-24]. Some testing results obtained during the Experiment are given in this article as an illustration.

## Results

**Figure 1.**
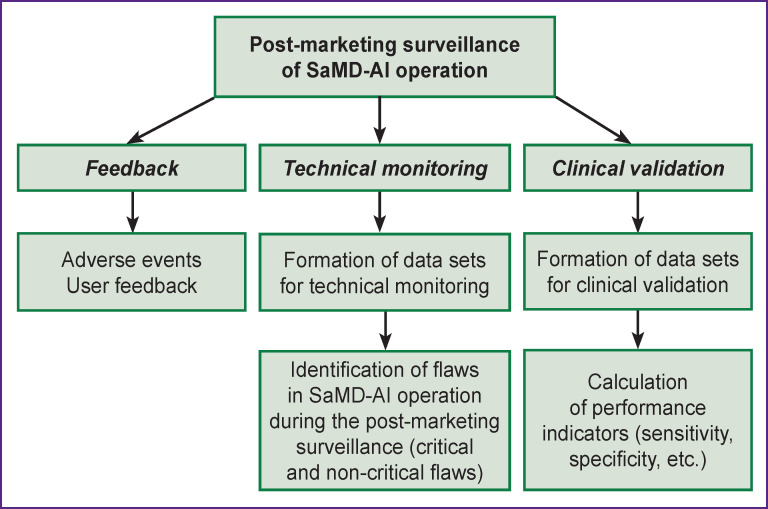
Key elements of the post-marketing surveillance of SaMD-AI

The PSM methodology proposed in this article for evaluating SaMD-AI performance is shown in Figure 1. The “Feedback” block includes the collection of data on adverse events, user feedback, which, among other things, can be collected by means of surveys [21]. This stage is common for all types of MD and its discussion in this regard is beyond the scope of this publication.

The analysis of the SaMD-AI safety and effectiveness during monitoring involves testing for technical monitoring and clinical validation using data sets.

### Technical monitoring

Technical monitoring is routine testing (either once per week or once per month), whose purpose is to monitor the quality of SaMD-AI performance for timely detection and elimination of technical flaws and the assessment of the product stability.

To perform this inspection, we used a pseudo-random sample of studies with the following breakdown: 25% of studies where SaMD-AI did not detect any pathology (“no pathology”), and 75% of studies contained a pathology (“contains pathology”). This distribution is due to the fact that the detection of pathological findings triggers additional functionality (i.e., the pathological area labelling). The selected studies with SaMD-AI results are reviewed by medical experts for technical flaws. The study is assigned a “contains pathology” status if the probability of having a pathology exceeds the optimal threshold value [24]. Otherwise, the study is considered as “having no pathology”.

Based on the results of a preliminary analysis of technical flaws in several SaMDs-AI participated in the Experiment, the main categories and subcategories of emerging errors were identified (see the Table). These are the main categories, since they affect the SaMD-AI safety and effectiveness during user operation, which includes image distortion in the SaMD-AI outputs, errors in SaMD-AI performance, and data processing timeout. The list has been supplemented with flaw grading (i.e., critical/non-critical). A flaw was considered critical if it makes their intended use practically impossible or unacceptable.

**Table:** List of technical flaws that may be observed during the post-marketing surveillance of SaMD-AI used in medical imaging

Type of flaw	Subtype of flaw (critical/non-critical)
1. Distorted images in the SaMD-AI results	1.1. Images are cropped in the region of interest (critical)
	1.2. The brightness/contrast of the image has been altered and cannot be corrected (critical)
	1.3. Labelling is outside a target organ (critical)
2. Errors in the results of SaMD-AI analysis/operation	2.1. No results from SaMD-AI (critical)
	2.2. Failure to analyze all images, an incorrect projection or series was analyzed (critical)
	2.3. No interpretation of findings (critical)
	2.4. Two contradicting interpretations in the results provided by the same SaMD-AI (critical)
	2.5. No warning label: “Study processed by SaMD-AI. Specialist confirmation required” (critical)
	2.6. Contradictory information in the interpretation of the image from SaMD-AI after processing (critical)
	2.7. Missing name or version of SaMD-AI (non-critical)
	2.8. SaMD-AI outputs contain no original series and images processed by SaMD-AI (if outlined in the technical documentation for SaMD-AI) (non-critical)
3. Exceeding the average analysis time	3.1. Image processing time is longer than the time spent by the physician (critical)

The Experiment established that the proportion of studies with technical flaws should not exceed 10% of the data set used in the ongoing testing. This requirement also complies with the provisions of clause 5.2 of GOST R ISO 2859-1-2007.

During the field testing of the technical monitoring procedure, the participants of the Experiment conducted more than 550 SaMD-AI tests. 60% of the tests were computed tomography, 28% — X-ray/photofluorography, and 12% — mammography.

The use of this methodology led to a gradual reduction in the number of studies with flaws due to timely improvements to artificial intelligence algorithms.

As a result, a 5% reduction in flaws was observed for various SaMDs-AI during subsequent testing.

**Figure 2.**
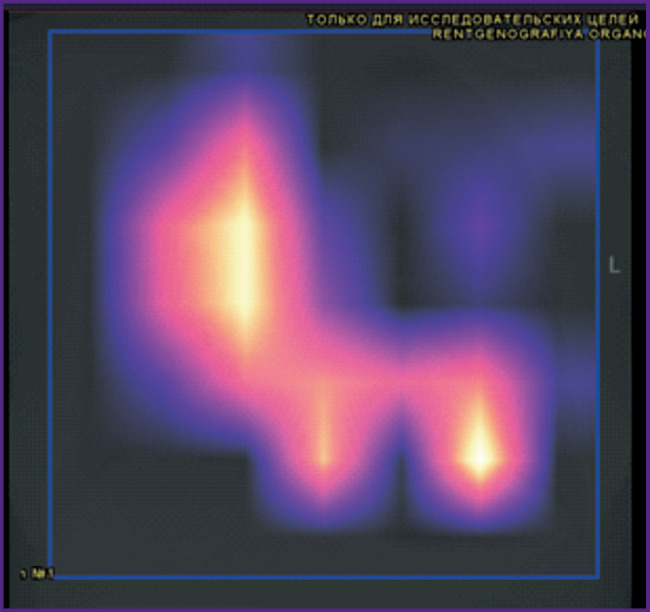
An example of SaMD-AI technical flaws (1.2 and 2.8, see the Table) using evidence from chest radiography

**Figure 3.**
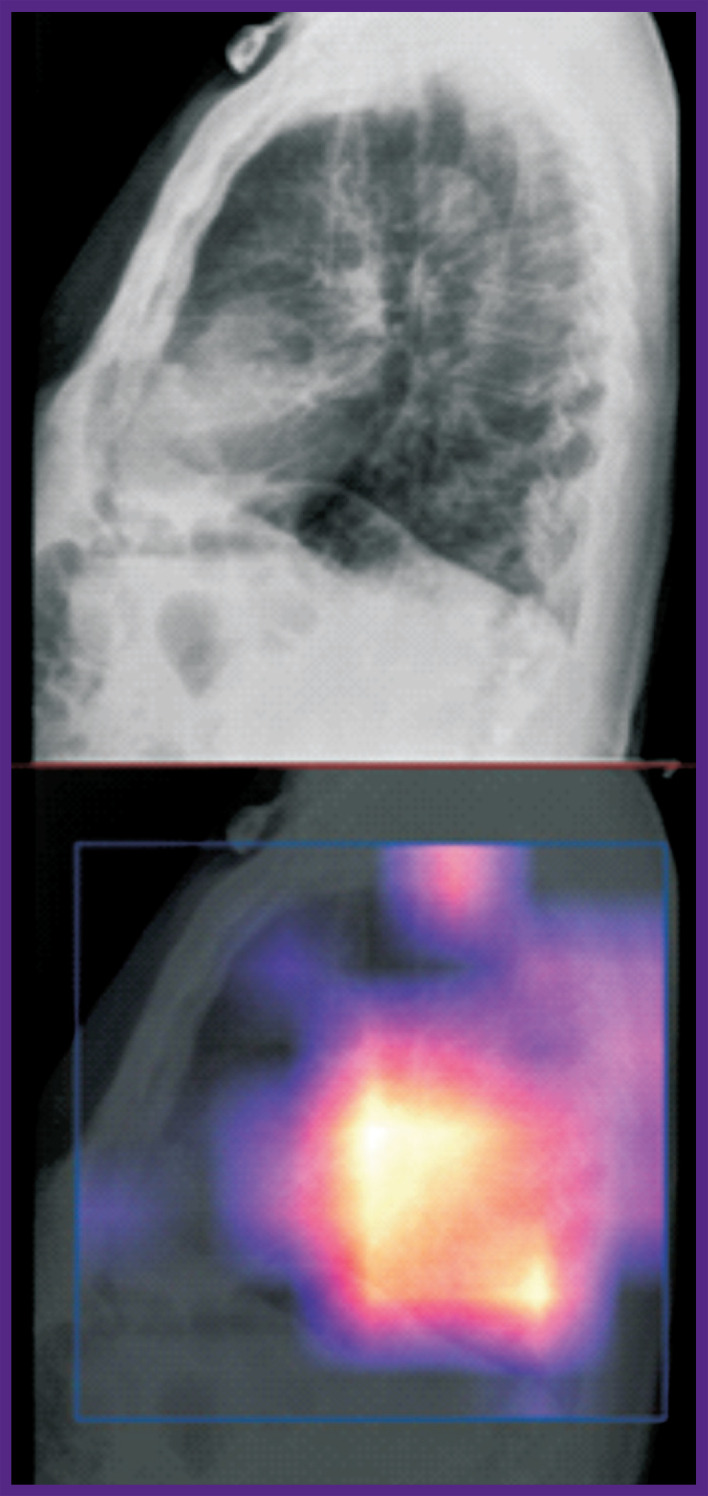
An example of an SaMD-AI technical flaw (2.2, see the Table) using evidence from chest radiography

**Figure 4.**
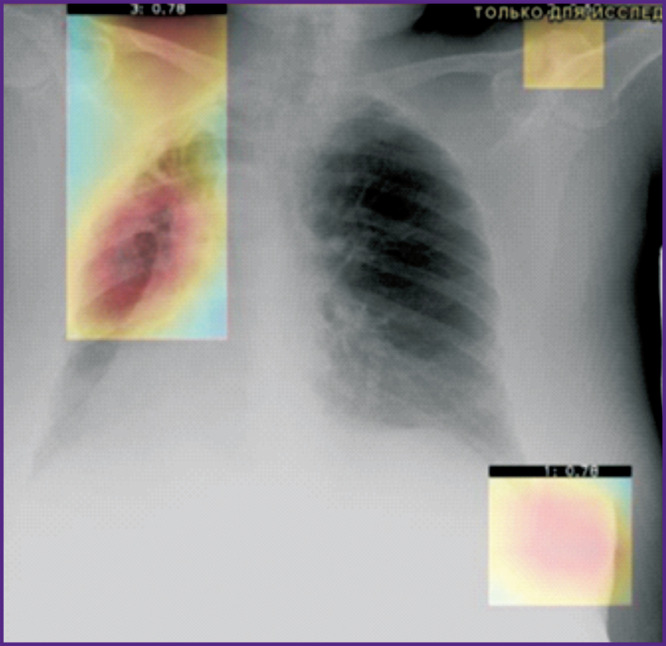
An example of an SaMD-AI technical flaw (1.3, see the Table) using evidence from chest radiography

**Figure 5.**
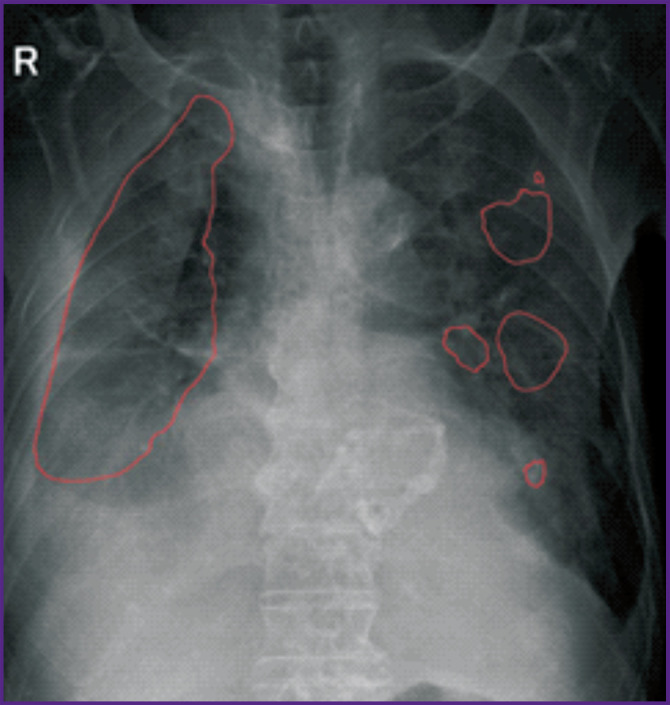
An example of SaMD-AI technical flaws (2.5 and 2.7, see the Table) using evidence from chest radiography

Let us consider the technical monitoring of SaMD-AI using the evidence from chest imaging studies. The results for four images presented in Figures 2–5 demonstrate the technical flaws detected during the SaMD-AI testing flaw (see the Table).

Figure 2 illustrates flaw 1.2 (The brightness/contrast of the image has been altered and cannot be corrected) and flaw 2.8 (SaMD-AI outputs contain no original series and images processed by SaMD-AI). The SaMD-AI has altered the original version and contrast of the original image so that the region of interest could be inspected, and it became impossible to identify the SaMD-AI results.

Figure 3 illustrates technical flaw 2.2 (Failure to analyze all images, an incorrect projection or series was analyzed). SaMD-AI processed and superimposed a heat map on the lateral view of the X-ray images. However, according to the functional purpose of SaMD-AI, correct processing is only possible for frontal projections.

Figure 4 illustrates technical flaw 1.3 (Labelling is outside a target organ). According to the performance results, SaMD-AI revealed findings outside the region of interest (i.e., the chest).

Figure 5 illustrates technical flaw 2.5 (No warning label: “Study processed by SaMD-AI. Specialist confirmation required”) and flaw 2.7 (Missing name or version of SaMD). The consequences of such flaws are as follows: the patient could see this image not knowing the results have been produced by SaMD-AI, and that they have neither been confirmed nor rejected by the reader, which could trigger confusion or excessive anxiety in the patient.

When analyzing the technical monitoring outcomes produced by the Experiment between September and November 2021, the average number of these flaws was 13% (standard deviation — 4.2%). At the same time, the prevailing flaws were “No interpretation of findings” (28%) and “Labelling is outside a target organ” (26%) which are critical.

### Clinical validation

**Figure 6.**
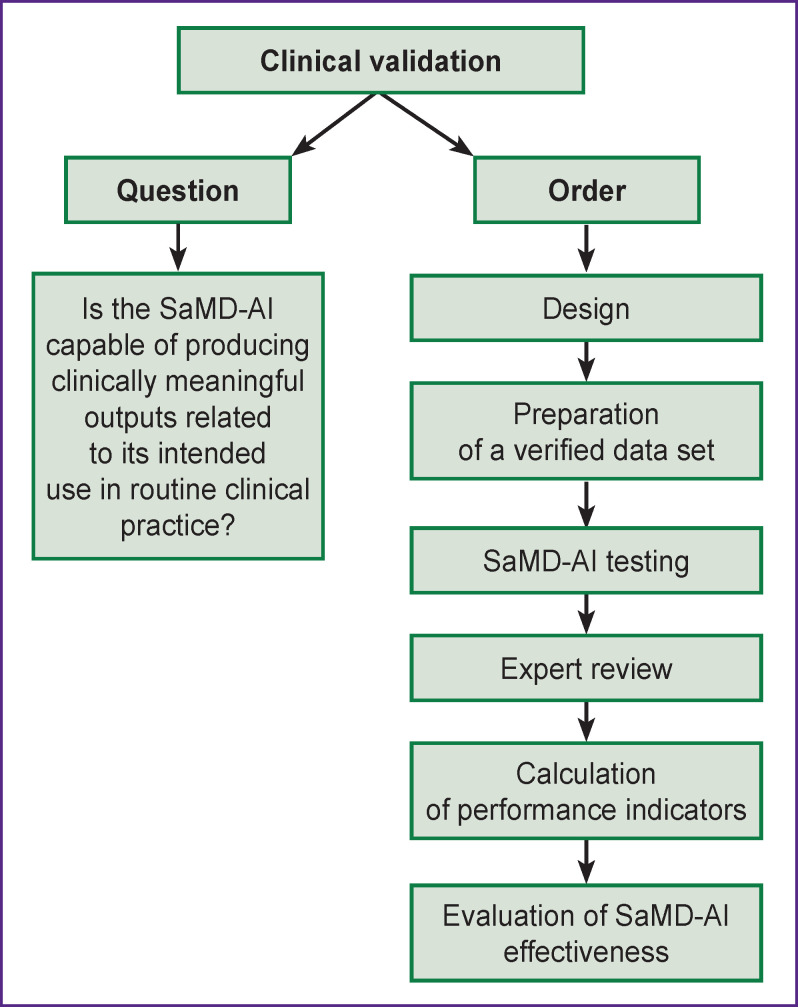
Procedure for performing clinical validation of SaMD-AI

Clinical validation confirms the SaMD-AI capability to provide clinically meaningful outputs related to its intended use within the functional purpose established by the developer (Figure 6) [17].

For each SaMD-AI, the performance parameters (i.e., sensitivity, specificity, accuracy, etc.) must be set and passed to the experts conducting the verification testing during PSM. The set of indicators that set the clinical validation framework vary depending on the purpose and functionality of SaMD-AI [23].

It is important to mention, that during the testing most of the time SaMD-AI, instead of definite pathological findings, yields a quantitative parameter that determines the probability of detecting a pathology on each particular image, ρ∈[0.1]. The presence of a pathology on the image is acknowledged if the value of ρ exceeds the specified threshold value *T*. The sensitivity, specificity, and the other evaluated SaMD-AI parameters depend on the selected *T* value.

When choosing the optimal threshold value, one should be guided by the clinical task this SaMD-AI is designed to solve. For example, clinical screening requires increased sensitivity, since the clinical task is to minimize the number of missed findings. For these purposes, we may recommend to calculate the threshold value by assessing the predictive value for negative results of diagnostic tests (maxNPV). However, with a threshold set in this way, the SaMD-AI may be of no use for clinicians that use it as a system to support clinical decision-making, which calls for a balance between sensitivity and specificity. In this case, it is recommended to use the threshold value determined by Youden index maximization [23, 25]. For narrow objectives associated with detecting pathologies, we suggest assessing the predictive value for positive results of diagnostic tests (maxPPV). It is also possible to calculate a threshold to set a certain sensitivity or specificity of SaMD-AI depending on specific conditions. The optimal threshold can be chosen by analyzing the characteristic curve (ROC curve). When analyzing different points on the curve, the threshold value corresponding to the highest value of the Youden index is chosen: Y=sen+spe–1, where sen is sensitivity, spe is specificity. After determining the optimal threshold, the performance metrics of SaMD-AI are calculated [26]. In order to standardize the outcomes, diagnostic accuracy metrics calculated for the Youden method are used.

#### Building a data set for clinical validation

Clinical validation of the SaMD-AI is carried out using data sets large enough to produce results with the claimed accuracy. This feature distinguishes verification from testing for the presence of technical flaws.

The size of the data set (i.e., the sample size) for evaluating the SaMD-AI characteristics is determined using proportional sampling and statistics (i.e. a method for establishing the required accuracy for estimated sensitivity and specificity claimed by the developer) [27, 28]. The data set building process is presented in detail in the papers [29-31].

1. *Defining goals and objectives*. The purpose of forming a data set must be determined. Only then it would be possible to assess whether the data or other data processing activity can be accessed:
  collectible data;
  intended use (i.e., specific tasks);
  disclosure regime (third-party access);
  data availability period.
  Before assigning a data set building task, one must define the subject area and the processing methods.
2. *Obtaining an approval of the ethics committee* (if necessary).
3. *Providing access to the data set*. The process of organizing access must be documented and the data (including personal data) protection procedures must be secured in compliance with the current legislation. The data transfer rate must match the goals and objectives of such access.
4. *Data collection* includes the delivery of clinical data (i.e., phenomena, syndromes, diseases, outcomes) according to their frequency of occurrence and incidence in the population (if determined by the purpose of the trials). The sample size and frequency of occurrence must be determined during the statistical estimation in accordance with the data set purpose.
5. *De-identification*. Any personal information must be removed from both the metadata and the source data.
6. *Structuring the data set*. The data dimensionality can be reduced.
7. *Filtration*. The data set filtering stage allows to reduce the cost of data labelling by ruling out those that do not match the specified parameters.
8. *Data labelling (annotation)*. Annotation types are presented in the papers [29, 30].
9. *Organization of storage and access to the verified data set*. Data storage can be organized on a local server or using cloud storage (GOST R ISO/ IEC 17826-2015).

#### An example of clinical validation

As part of the work of the Research and Practical Clinical Center for Diagnostics and Telemedicine Technologies, the Sector for Clinical and Technical Trials has tested the developed PSM methodology, which includes clinical validation of SaMD-AI.

The first stage of clinical validation is the formation of a verified data set, i.e., a set with confirmed clinical data.

In order to calculate the ratios between the images with and without pathological findings in the data set (samples), the former must be singled out to check for the claimed sensitivity, while the images that do not contain pathologies (signs) indicated in the developer’s documentation are selected to assess specificity.

In order to build the initial data set, we used clinical data from the Unified Radiological Information Service of the Unified Medical Information and Analytical System of the city of Moscow (URIS/UMIAS). The data were filtered using the following parameters: procedure name, type of diagnostic device, type of medical organization, date, age of the patient. We used data from 18 different models of diagnostic devices, 4 of which were fluorography devices (670 studies), the remaining 14 being X-ray diagnostic devices (866 studies). The conclusions of radiologists were also uploaded from the URIS/UMIAS for the selected studies. The generated preliminary data set was visually analyzed by the radiologists from the research team. The analysis ruled out:

controversial images that do not have obvious pathologies listed in the developer’s documentation and which cannot be reliably categorized as “normal”;

images with insufficient technical quality (low contrast, etc.).

Verification of the generated data set was carried out by the radiologists from the group of researchers who have academic degrees and experience in this field. In addition, the procedures (incl. de-identification) were performed according to the algorithm given earlier.

The second stage of clinical validation is the evaluation of SaMD-AI performance with a verified data set. It was carried out using Python software. We calculated sensitivity and specificity of SaMD-AI and compared the calculated values with the accuracy indicators claimed by the SaMD-AI developer.

The analysis carried out during clinical validation made it possible to confirm the characteristics claimed by the developer and draw reliable conclusions about the effectiveness and safety of SaMD-AI.

## Discussion

The PSM methodology proposed in this article provides means to monitor the safety and effectiveness of SaMD-AI in accordance with the established regulatory requirements and the specifics of SaMD-AI as an innovative product.

The existing regulatory documents provide general requirements for such monitoring and necessitate the development of specific requirements applicable to SaMD-AI. The developed PSM scheme for SaMD-AI meets the global requirements for verification and testing of MD for regulatory compliance with due regard to SaMD-AI features. Thus, the paper by Park et al. [32] states, that it is important to conduct both testing for the presence of technical flaws and the assessment of routine clinical practice. The steps outlined in this article ensure comprehensive monitoring of SaMD-AI that meets all the requirements of the methodology presented in the paper [32].

In their paper, Benjamens et al. [9] raised concerns associated with rapid updating SaMD-AI as an intangible MD and, accordingly, extension of SaMD-AI re-registration timeline. According to the FDA, this type of medical device requires a separate registration system that addresses the full life cycle of the product. The methodology must allow to introduce changes during adaptation and operation of SaMD-AI, while ensuring its safety and effectiveness as an MD [11]. The article by Kelly et al. [10] discusses the steps necessary to ensure the safe, but also rapid registration of SaMD-AI, and the introduction of these products into real-world clinical practice. The authors note, that it is important and necessary to evaluate SaMD-AI performance using real clinical data and compare it with the previous assessments in order to eliminate possible scatter of SaMD-AI features. The SaMD-AI developers must be vigilant of potential hazards, including challenges of using new patient populations and unintended negative health outcomes. It is necessary to analyze both the main performance indicators (metrics) of SaMD-AI (the area under the characteristic curve), and predictive indicators, i.e., positive and negative predictive values.

In addition to the above FDA recommendations regarding SaMD-AI monitoring, the Guidance from the Health Sciences Authority of Singapore has recently been released [33]. The document mandates, that SaMD-AI must carry out collection and analysis of data from routine clinical practice, and submit the results to the regulatory body at regular intervals. The European Union has also approved a document that includes similar requirements for monitoring of any medical software [2]. It is necessary to periodically collect and analyze data to assess the safety and effectiveness of the product in real practice, as well as to analyze the feedback. Thus, there is a tendency to follow the same requirements to PSM both with the use of medical software in general (as noted in the European Union document), and SaMD-AI in particular, as specified by the FDA and the Health Sciences Authority of Singapore. Our paper also provides a classification of possible errors in SaMD-AI used during technical monitoring, which is a more advanced version of the proposed scientific approaches.

The importance of the technical monitoring of SaMD-AI, i.e., software as MD, has been emphasized in several FDA publications. Changes can be made to the software after it has been registered as an MD [34]. According to the FDA statistics, 79% of identified software errors at the post-registration stage are attributed to the changes made to it [18]. It is also noted, that most of the software errors associated are observed in medium-risk MD [35]. The methodology for systematizing SaMD-AI flaws presented in this article is unique, since the reviewed sources do not provide detailed descriptions of possible flaws that may be observed during SaMD-AI operation. The grouping of SaMD-AI flaws into critical and non-critical provided in this paper have substantial practical value. This can be used by both SaMD-AI software developers and users when assessing the presence of adverse events that should be taken into account during PSM reporting and compiling and evaluating the user feedback.

The proposed PSM approaches can be implemented to monitor the safety and efficiency of SaMD-AI by regulatory authorities, and to strengthen the quality management system of the developers. ISO/TR 20416:2020 [36] recommends linking the PSM plan into all the processes within the SaMD-AI quality control framework (risk management, clinical evaluation, etc.). In order to ensure the traceability of the SaMD-AI results, it is also important to analyze and compare the results of the current PSM with previous data [37], monitor performance trends and feedback parameters, and carry out technical monitoring to make timely corrections and ensure safety and efficiency of SaMD-AI software.

The methodology presented in this article can be used by SaMD-AI developers during the making and implementing of an PSM plan, which must be submitted as part of the set of documents for registration of medical devices. While the Eurasian Economic Commission mandates SaMD-AI developers to carry out such monitoring and submit reporting to the regulatory authorities within 3 years, the FDA recommends that such monitoring should be conducted throughout the entire product life cycle.

The examples given in this paper relate to the field of diagnostic radiology; however, the described PSM methodology can be applied to SaMD-AI used in any field of clinical medicine that utilizes clinical data [29, 31]. Changes will mainly be required in the formation of a list of technical flaws and their classification, since this information will be specific to each field of medicine.

The required stages of PSM for SaMD-AI operation from the methodology shown in the article are presented in a series of national standards devoted to clinical medicine [30, 38–41]: these documents provide a PSM plan, and the goals and objectives adapted specifically for SaMD-AI.

## Conclusion

The post-marketing surveillance is regulated by the current regulatory legal acts; however, the requirements, as a rule, are established for medical devices in general. The proposed methodology for the post-marketing surveillance of the SaMD-AI using evidence from diagnostic imaging includes feedback assessment, technical monitoring for the presence of flaws in the SaMD-AI operation, and the clinical validation of such software for effectiveness. The implementation of this monitoring will ensure the safety and efficiency of the SaMD-AI with regard to the specifics of these products as non-material medical devices.
